# LRRC8 N termini influence pore properties and gating of volume-regulated anion channels (VRACs)

**DOI:** 10.1074/jbc.RA118.002853

**Published:** 2018-06-20

**Authors:** Pingzheng Zhou, Maya M. Polovitskaya, Thomas J. Jentsch

**Affiliations:** From the ‡ Leibniz-Forschungsinstitut für Molekulare Pharmakologie (FMP) and Max-Delbrück-Centrum für Molekulare Medizin (MDC), D-13125 Berlin, Germany,; ¶ Neurocure Cluster of Excellence, Charité Universitätsmedizin, D-10117 Berlin, Germany, and; § Graduate Program, Faculty of Biology, Chemistry, and Pharmacy, Freie Universität Berlin, D-14195 Berlin, Germany

**Keywords:** ion channel, chloride channel, biophysics, electrophysiology, membrane transport, ICl,swell, ICl,vol, swelling-activated chloride channel, VSOAC, VSOR, amino-terminus, N-terminus

## Abstract

Volume-regulated anion channels (VRACs) are crucial for cell volume regulation and have various roles in physiology and pathology. VRACs were recently discovered to be formed by heteromers of leucine-rich repeat–containing 8 (LRRC8) proteins. However, the structural determinants of VRAC permeation and gating remain largely unknown. We show here that the short stretch preceding the first LRRC8 transmembrane domain determines VRAC conductance, ion permeability, and inactivation gating. Substituted-cysteine accessibility studies revealed that several of the first 15 LRRC8 residues are functionally important and exposed to a hydrophilic environment. Substituting glutamate 6 with cysteine decreased the amplitudes of swelling-activated *I*_Cl,vol_ currents, strongly increased iodide-over-chloride permeability, and markedly shifted the voltage dependence of channel inactivation. Importantly, these effects were reversed by 2-sulfonatoethyl methanethiosulfonate, which restores the negative charge at this amino acid position. Cd^2+^-mediated blocking of *I*_Cl,vol_ in cysteine variants suggested that the LRRC8 N termini come close together in the multimeric channel complex and might form part of the pore. We propose a model in which the N termini of the LRRC8 subunits line the cytoplasmic portion of the VRAC pore, possibly by folding back into the ion permeation pathway.

## Introduction

Maintenance of a constant volume upon changes in extracellular or intracellular osmolarity is critical for the function and survival of cells (1). After swelling, cells readjust their volume in a process called regulatory volume decrease, which involves the regulated passive efflux of chloride, potassium, and small organic compounds that osmotically drive the outflow of water. A key player in regulatory volume decrease is the volume-regulated anion channel (VRAC),^4^ which is also known as volume-sensitive outwardly rectifying anion channel (VSOR) (2) or volume-sensitive organic osmolyte-anion channel (VSOAC) (3). VRACs seem to be ubiquitously expressed in vertebrate cells. They not only transport chloride but also various molecules, including organic osmolytes, neurotransmitters, and drugs (1, 4–7). Besides regulating cell volume, VRAC may play roles in signal transduction, cell migration, apoptosis, tumor drug resistance, and stroke (1, 5, 8).

VRAC currents (named *I*_Cl,vol_ or *I*_Cl,swell_) were first reported nearly 30 years ago (9, 10) and have been extensively characterized since then (11, 12). VRACs are largely closed under resting conditions. Their opening by cell swelling, which may involve several poorly understood mechanisms, leads to typical anion currents that display an SCN^−^ > I^−^ > NO_3_^−^ > Br^−^ > Cl^−^ > F^−^ permeability sequence, variable inactivation at cytoplasmic positive potentials, and moderate outward rectification (1, 11–13).

Our ignorance of the proteins mediating *I*_Cl,vol_ (2, 14) has precluded structure–function analyses of VRAC until recently when the channel was found to be composed of heteromers of LRRC8 proteins (4). LRRC8A, the only essential VRAC subunit (4, 15), needs at least one of the other proteins (LRRC8B, -C, -D, or -E) encoded by the *LRRC8* gene family to form functional channels (4). Instead of a single VRAC, there are a large number of differently composed VRACs, taking into account that LRRC8 proteins assemble to hexamers (4, 16, 17) or higher-order oligomers. These channels may differ in inactivation (4, 18), permeation of organic substrates (5, 6, 19), single-channel conductance, and rectification (17). The physiological importance of LRRC8/VRAC channels is underscored by the high lethality and multiple tissue abnormalities of *Lrrc8a*^−/−^ mice (20) and their role in, for instance, apoptosis and tumor drug resistance (5) and β-cell insulin secretion (22, 23).

LRRC8 proteins have four predicted transmembrane domains that are followed by a large hydrophilic segment containing 16 leucine-rich repeats (24) (hence LRRC for leucine-rich repeat–containing). Analysis of databases (16) and experiments with transfected cells (4, 7, 15) indicated that both N and C termini of LRRC8 proteins face the cytoplasm. This finding agrees with the sequence homology of the LRRC8 transmembrane region to pannexin and innexin channels (16). These three related protein families are believed to share their protein fold with gap junction–forming connexins (16) and CALHM channels (25) even though they display no significant sequence homology. Indeed, cryo-EM revealed a connexin-like protein fold for *Caenorhabditis elegans* innexin-6 (26) and most recently also for LRRC8 channels (27).

The structural determinants of VRAC function remain largely unknown. Chimeras between the poorly inactivating LRRC8C and the rapidly inactivating LRRC8E isoforms were used to pinpoint residues involved in the voltage-dependent inactivation of VRAC (18). Mutating some of these residues (*e.g.* Lys-98), which are located in the C terminal portion of the first extracellular loop (EL1), also mildly increased the I^−^/Cl^−^ permeability ratio of LRRC8A/E heteromers (18), and the LRRC8A R103A mutant increased cation permeability of LRRC8A/C heteromers (27). Small changes in the iodide/chloride permeability ratio (*P*_I_/*P*_Cl_) were also observed when Thr-44, located at the end of the first transmembrane span, was mutated to cysteine (15). These residues might therefore participate in forming the external portion of the pore as recently confirmed by the cryo-EM structure of LRRC8 channels (27).

In the course of previous studies (4), we noticed that the addition of epitopes to the N terminus of LRRC8A abolished VRAC currents. This observation suggested an important role of LRRC8 N termini in channel function. In this work, we therefore extensively mutated N-terminal residues of LRRC8A and LRRC8C, coexpressed both subunits, and examined swelling-activated *I*_Cl,vol_ currents. Cysteine modification experiments and effects on permeation and gating suggest that the N termini of LRRC8 proteins participate in forming the cytoplasmic portion of the VRAC pore.

## Results

**Figure 1.**
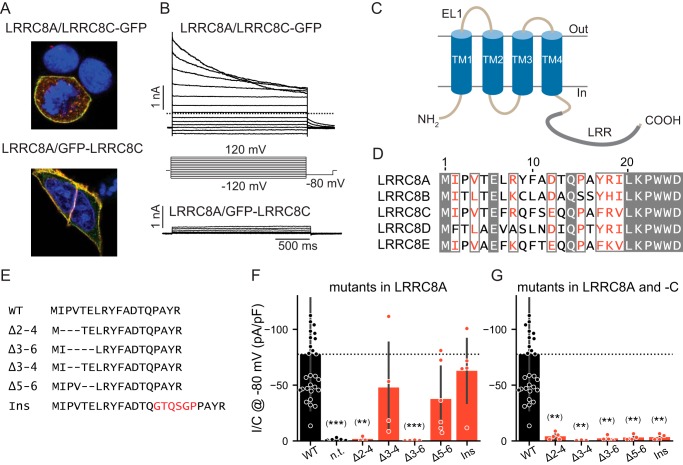
**Modifications of LRRC8 N termini interfere with channel function.** *A*, plasma membrane localization of LRRC8A and LRRC8C-GFP or GFP-LRRC8C upon coexpression in *LRRC8*^−/−^ HCT116 cells. LRRC8A was detected by LRRC8A antibody (*red*), and LRRC8C-GFP (*top*) or GFP-LRRC8C (*bottom*) was detected by a GFP antibody (*green*). *Yellow* color indicates colocalization of LRRC8A and -C. *B*, typical current traces obtained with of LRRC8C-GFP (*top*) or GFP-LRRC8C (*bottom*) upon coexpression with LRRC8A and >8-min exposure to 25% hypotonic solution (240 mosm). *C*, topology model of LRRC8 proteins with four TMs and cytoplasmic LRRs (indicated by *thick gray line*). *D*, amino acid sequence alignment of N termini from human LRRC8A–E proteins. Conserved residues are shown in *white letters* on *gray background*, and homologous amino acids are displayed in *red*. The first transmembrane span begins at about Trp-23. *E*, sequence of generated N-terminal deletion and insertion (*Ins*) mutants of LRRC8A aligned to the WT sequence (*top*). The arbitrary uncharged and flexible peptide was inserted after Gln-14 because the N termini of connexin 26 make a kink at Asn-14 to dip into the pore (31). *F* and *G*, current densities of hypotonicity-stimulated *I*_Cl,vol_ at −80 mV of WT LRRC8A deletion/elongation mutants coexpressed with WT LRRC8C (*F*) or with LRRC8C carrying equivalent deletions/insertions (*G*). *n.t.*, nontransfected controls. *Error bars*, S.D.; **, *p* < 0.01; ***, *p* < 0.001 *versus* WT (Dunn's post hoc test after Kruskal–Wallis test; *p* values are corrected using the Benjamini–Hochberg procedure). *pF*, picofarad.

Cotransfection of *LRRC8*^−/−^ HCT116 cells (in which all five *LRRC8* genes have been disrupted (4)) with LRRC8C and either green florescent protein (GFP)-LRRC8A or LRRC8A-GFP or with LRRC8A and either GFP-LRRC8C or LRRC8C-GFP resulted in fluorescence at the outer cell membrane (Fig. 1*A* and Fig. S1, *A–C*). Detection of GFP-tagged LRRC8C at the cell surface suggested the formation of LRRC8A/C heteromers because LRRC8C needs LRRC8A for plasma membrane expression (4). However, only cells expressing the construct in which the GFP had been fused to the C terminus rather than the N terminus of LRRC8A (Fig. 1*B*) or LRRC8C (Fig. S1, *Y* and *Z*) yielded swelling-activated *I*_Cl,vol_ currents. Currents elicited by LRRC8A-GFP/LRRC8C or LRRC8A/LRRC8C-GFP were indistinguishable and displayed typical slow inactivation of LRRC8A/C currents as reported previously (4, 18). Because N-terminal addition of GFP to LRRC8A or LRRC8C interfered with ionic currents but not protein localization, LRRC8 N termini might be critically involved in forming or regulating the pore of VRAC channels.

All LRRC8 isoforms display short, ∼18-amino-acid-long N termini before the first predicted transmembrane domain (Fig. 1, *C* and *D*). In addition to the obligatory initiator methionine, Glu-6 and Gln-14 are conserved in all subunits, whereas other parts of the N terminus display significant homology (Fig. 1*D*). We cotransfected various N-terminal deletion or elongation mutants of LRRC8A (Fig. 1*E*) with LRRC8C, either WT or carrying the same deletion or insertion (with GFP fused to the C terminus of LRRC8C for identification of transfected cells as in all subsequent experiments), into *LRRC8*^−/−^ HCT116 cells. When coexpressed with WT LRRC8C, LRRC8A deletions Δ2–4 and Δ3–6 abolished *I*_Cl,vol_, whereas Δ3–4 and Δ5–6 and the insertion of 6 additional amino acids after Gln-14 were tolerated (Fig. 1*F*). However, when present in both isoforms, the latter deletions and insertions also abolished currents (Fig. 1*G*). In all cases, LRRC8C-GFP was detected at, or close to, the plasma membrane (Fig. S1, *E–N*). Because LRRC8A is required for the surface expression of LRRC8C (4), this localization suggested that these deletions and the insertion did not interfere with the assembly of LRRC8A/C heteromers. However, we cannot exclude that the decrease in current amplitudes with some of the mutants is due, at least in part, to decreased surface expression.

### Cysteine scanning of LRRC8 N termini and modification by 2-aminoethyl methanethiosulfonate (MTSEA) and Cd^2+^

**Figure 2.**
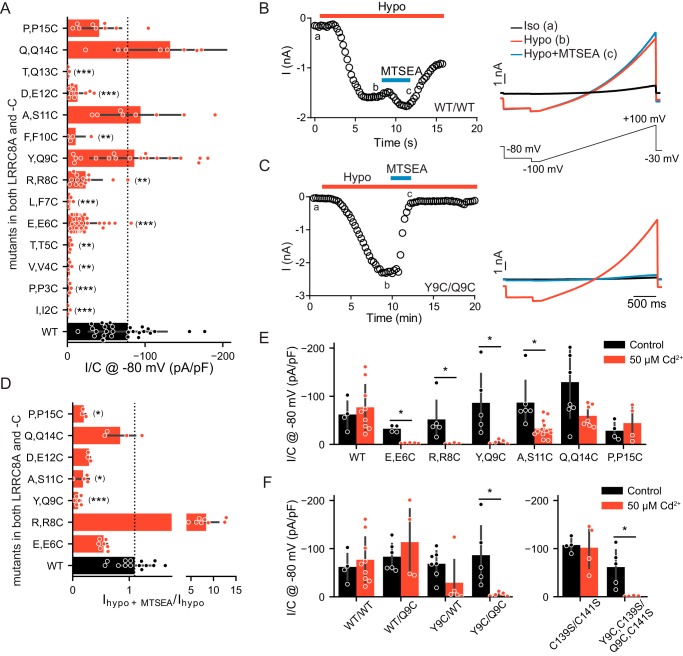
**Cysteine-scanning mutagenesis of LRRC8A/C N termini.** WT and mutated LRRC8A and -C were coexpressed in *LRRC8*^−/−^ HCT116 cells, and *I*_Cl,vol_ were elicited by hypotonicity as in Fig. 1*B. A*, mean maximum *I*_Cl,vol_ current densities (current normalized to respective cell capacitance) of LRRC8A/C channels carrying cysteine substitutions in both LRRC8A and -C. Mutations are indicated on the *vertical axis* with native residues in LRRC8A and -C separated by *commas*. The *dashed line* indicates mean *I*_Cl,vol_ density from WT/WT LRRC8A/C channels (WT measurements identical to Fig. 1*F*). *B* and *C*, typical effects of MTSEA on *I*_Cl,vol_ mediated by WT/WT (*B*) or Y9C/Q9C (*C*) LRRC8A/C channels. *Left*, representative time course of currents at −80 mV taken from voltage ramps applied every 15 s. Each *circle* represents an individual measurement. *Red* and *blue bars* indicate application of 25% hypotonic solution and hypotonic solution containing 200 μm MTSEA, respectively. *Right*, typical current traces elicited by these ramps (shown below the *upper panel*) in isotonic solution (*Iso*; *a*), full activation by hypotonic solution (*Hypo*; *b*), and at the end of the ∼ 4-min perfusion of MTSEA (*c*). Capacitance of the cells in *B* and *C*: 18.2 and 18.8 picofarads (*pF*), respectively. *D*, mean effect of MTSEA on maximal *I*_Cl,vol_ currents of LRRC8A/C heteromers carrying cysteine mutations in both LRRC8A and -C. Currents are normalized to those before MTSEA application. The *dashed line* represents mean, normalized current density of MTSEA-exposed WT/WT channels. *E*, effect of 50 μm intracellular Cd^2+^ (applied in pipette solution) on *I*_Cl,vol_ density from WT and mutant LRRC8A/C heteromers. *F*, effect of 50 μm intracellular Cd^2+^ (applied in pipette solution) on *I*_Cl,vol_ density from WT and mutant LRRC8A/C heteromers (the values for WT and Y9C/Q9C are the same as in *E*) and for LRRC8A^C139S^/C^C141S^ with and without Y9C/Q9C mutation. *Error bars*, S.D.; *, *p* < 0.05; **, *p* < 0.01; ***, *p* < 0.001 *versus* WT (in *A* and *D*, Kruskal–Wallis test, Dunn's post hoc test; in *E* and *D*, Mann–Whitney test, false-discovery rate controlled by Benjamini–Hochberg procedure).

We then systematically mutated N-terminal residues in LRRC8A and LRRC8C and assessed swelling-activated currents after coexpressing both subunits in *LRRC8*^−/−^ HCT116 cells (4). To explore the general validity of our findings, we also studied several equivalent mutations in LRRC8D and -E. Initially we replaced N-terminal residues by cysteines because changes in channel properties by cysteine-modification reagents may reveal the functional importance of the respective residue and its exposure to a hydrophilic environment (28). We singly replaced all residues from Ile-2 to Pro-15 in both LRRC8A and LRRC8C by cysteines and coexpressed the LRRC8A mutants with either WT LRRC8C or LRRC8C mutated at the equivalent position. Whereas 10 of 14 LRRC8A mutants yielded currents when coexpressed with WT LRRC8C (Fig. S2*A*), only seven mutations gave functional channels when introduced into both isoforms (Fig. 2*A*). Of note, none of the cysteine mutants of the most N-terminal residues (positions 2, 3, and 4) gave currents.

We next explored the effect of the partially positively charged, cell membrane–permeable cysteine-modification reagent MTSEA (28) on currents of all functional “double mutants” (*i.e.* LRRC8A/C heteromers carrying the same mutation in both subunits) and on those functional “single” LRRC8A mutants that gave currents only when coexpressed with WT LRRC8C. Control experiments on WT LRRC8A/C–transfected cells gave slow, variable effects on *I*_Cl,vol_ amplitudes that sometimes included an initial current enhancement followed by a slow decrease to roughly 75% of the previous amplitude (Fig. 2, *B* and *D).* By contrast, currents from several cysteine mutants were strongly and rapidly reduced by MTSEA application (Fig. 2, *C* and *D*, and Fig. S2*B*). This effect persisted following the washout of the reagent (Fig. 2*C*), suggesting that it was caused by a covalent reaction of the engineered cysteine with MTSEA. The blocking effect of MTSEA was particularly striking with Y9C/Q9C channels, which were almost completely and irreversibly inhibited by MTSEA (Fig. 2, *C* and *D*). Interestingly, exposure of the R8C double mutant to MTSEA, which may restore a positive charge at position 8, strongly increased *I*_Cl,vol_ to values that even exceeded WT currents severalfold (Fig. 2*D* and Fig. S2*C*).

Cd^2+^ inhibition of cysteine-substituted channels can demonstrate the close vicinity of the substituted residues because Cd^2+^ binding requires multiple cysteines (29, 30). We had to apply Cd^2+^ to the N-terminal cysteine mutants from the intracellular side, that is from the pipette. Therefore, inhibition of VRAC may occur before or during its activation by cell swelling, precluding observations of acute *I*_Cl,vol_ inhibition. Indeed, with 50 μm Cd^2+^ in the pipette, hypotonicity no longer elicited *I*_Cl,vol_ from E6C/E6C, R8C/R8C, or Y9C/Q9C LRRC8A/C double mutants (Fig. 2*E*). By contrast, currents from A11C/S11C, Q14C/Q14C, and P15C/P15C mutant LRRC8A/C heteromers were much less affected (Fig. 2*E*). Cd^2+^ binding may occur between the newly introduced cysteines, which are located at equivalent positions, either between homologous (*i.e.* between LRRC8A and -C) or the same type of subunits (*e.g.* between two LRRC8A subunits). We therefore tested whether cysteines needed to be inserted into both LRRC8 isoforms of the heteromeric channel. Whereas WT/Q9C channels were unaffected by intracellular Cd^2+^, Y9C/WT channels were efficiently blocked by Cd^2+^ (Fig. 2*F*), suggesting that Cd^2+^ can be coordinated by cysteines of identical isoforms. Assuming that the N termini of LRRC8 proteins fold back into the pore as in connexin26 (31) and innexins (26), we considered the possibility that the newly introduced cysteines might coordinate Cd^2+^ together with transmembrane domain 2 (TM2) residues Cys-139 and Cys-141 in LRRC8A and LRRC8C, respectively, that localize to, or close to, VRAC's permeation pathway (27). Mutating both cysteines to serines resulted in functional LRRC8A/C channels as did N-terminal Y9C and Q9C mutations inserted in LRRC8A^C139S^/LRRC8C^C141S^ channels to yield LRRC8A^Y9C,C139S^/LRRC8C^Q9C,C141S^ channels (Fig. 2*F*). The latter channels were efficiently blocked by Cd^2+^ (Fig. 2*F*), demonstrating that the inhibition of *I*_Cl,vol_ is not due to a cross-link of N termini to the interior of VRAC. However, we cannot strictly exclude that Cd^2+^ binding occurred between the newly introduced and other cysteines present in cytoplasmic parts of the channel that appear to have variable conformations (27). This possibility might be rigorously eliminated only if all cytosolic cysteines were replaced by other residues in both subunits. Unfortunately, such cysteine-less mutants of LRRC8A and LRRC8C failed to yield currents (data not shown). In any case, the sum of our data suggests that the extreme N termini of LRRC8 proteins come close together in the multimeric channel complex and might form part of the pore.

### Mutations in Glu-6 change VRAC's halide permeability

**Figure 3.**
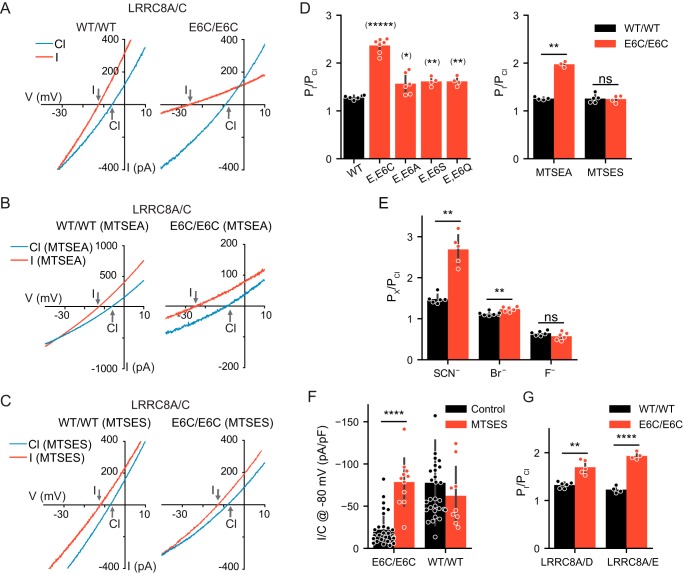
**Mutations of Glu-6 alter anion permeability of LRRC8 channels.** *A–C*, typical *I*_Cl,vol_ *I*/*V* curves (elicited by voltage ramps from −100 to +100 mV as in Fig. 2*B*) of WT/WT (*left*) and E6C/E6C (*right*) LRRC8A/C heteromers with 110 (*blue*) or 5 mm Cl^−^ and 105 mm I^−^ (*red*) extracellular Cl^−^. Note the large increase in iodide permeability in E6C/E6C mutants (*A*) that is not abolished by MTSEA (*B*) but is abolished by intracellular MTSES (1 mm) (*C*). *D*, *P*_I_/*P*_Cl_ obtained from shifts in reversal potentials for WT/WT and E6C/E6C, E6A/E6A, E6S/E6S, and E6Q/E6Q LRRC8A/C channels (*left*). Intracellular MTSES (1 mm) restored WT permeability ratio (*right*). *E*, mean permeability ratios (*P_X_*/*P*_Cl_) for SCN^−^, Br^−^, and F^−^ for WT/WT and E6C/E6C LRRC8A/C channels. *F*, restoration to WT levels of *I*_Cl,vol_ densities of E6C/E6C LRRC8A/C channels by intracellular MTSES (*left*), which lacked an effect on WT/WT channels (*right*) (control measurements as in Figs. 1*F* (WT) and 2*A* (E6C)). *G*, *P*_I_/*P*_Cl_ of WT/WT and E6C/E6C in LRRC8A/D and LRRC8A/E heteromers. *Error bars*, S.D.; *, *p* < 0.05; **, *p* < 0.01; ****, *p* < 0.0001; *****, *p* < 0.00001; *ns*, not significant (in *D*, *E*, and *G*, unequal-variance *t* test for pairwise comparisons; in *F*, Mann–Whitney test; false-discovery rate controlled by Benjamini–Hochberg procedure). *pF*, picofarad.

Mutations in pore-lining residues may change the selectivity of the permeation pathway. We therefore examined whether the functional LRRC8A/C heteromers carrying mutations either in LRRC8A or in both isoforms (Fig. 2*A* and Fig. S2) displayed altered I^−^/Cl^−^ permeability ratios, which were calculated from the shift of reversal potentials (Δ*E*_rev_) using the Goldman–Hodgkin–Katz equation (Table 1). Whereas most mutants had no or only marginal effects on VRAC's I^−^/Cl^−^ ion permeability, mutating Glu-6 to Cys in both LRRC8A and LRRC8C (LRRC8A^E6C^/C^E6C^ channels) dramatically increased *P*_I_/*P*_Cl_ from 1.29 ± 0.01 to 2.29 ± 0.10 (Fig. 3, *A* and *D*). Concomitantly the E6C/E6C mutation strongly decreased iodide conductance (Fig. 3*A*), suggesting an increased affinity of iodide to the mutant channel's pore. This double mutation also strongly increased SCN^−^ permeability while having only modest effects on Br^−^ and none on F^−^ permeability and leaving the selectivity sequence intact (Fig. 3*E*). Mutating Glu-6 in both LRRC8A and -C to alanine, serine, or glutamine also increased *P*_I_/*P*_Cl_, albeit less so (Fig. 3*D*). Average magnitudes of their *I*_Cl,vol_ were small, and E6K/E6K heteromers failed to yield measurable currents (Fig. S3B).

**Table 1:** **ΔE_rev_ and derived P_I_/P_Cl_ for cysteine mutants of LRRC8A/C channels**

Mutants	Δ*E*_rev_	*P*_I_/*P*_Cl_	*n*
	*mV*		
WT/WT	−5.90 ± 0.24	1.29 ± 0.01	5
T5C/WT	−7.13 ± 1.80	1.35 ± 0.10	4
E6C/E6C	−19.90 ± 1.04	2.29 ± 0.10	7
L7C/WT	−4.92 ± 0.85	1.23 ± 0.04	4
R8C/R8C	−9.10 ± 0.82	1.47 ± 0.05	3
Y9C/Q9C	−9.24 ± 0.40	1.48 ± 0.02	3
A11C/S11C	−7.2 ± 0.90	1.36 ± 0.05	3
D12C/E12C	−8.03 ± 0.53	1.41 ± 0.03	4
T13C/WT	−6.6 ± 1.20	1.30 ± 0.06	3
Q14C/Q14C	−4.92 ± 0.85	1.23 ± 0.04	4
P15C/P15C	−7.38 ± 0.57	1.37 ± 0.03	4

The positively charged MTSEA reduced the magnitude of the increase in I^−^/Cl^−^ permeability of the E6C double mutant (Fig. 3, *B* and *D*), whereas adding the membrane-impermeable (28), negatively charged 2-sulfonatoethyl methanethiosulfonate (MTSES) to the intracellular solution changed *P*_I_/*P*_Cl_ back to WT values (Fig. 3, *C* and *D*). Strikingly, MTSES not only restored the ion selectivity but also the current amplitude back to WT levels (Fig. 3*F*), stressing the importance of a negative charge at this position. Currents from LRRC8A^E6C^/C^E6C^ channels were efficiently blocked by 4-[(2-butyl-6,7-dichloro-2-cyclopentyl-2,3-dihydro-1-oxo-1*H*-inden-5-yl)oxy]butanoic acid (DCPIB) both in the absence and presence of MTSES, showing that other properties such as inhibitor sensitivity were preserved in these channels (Fig. S4*A*). Interestingly, MTSES had virtually identical effects on LRRC8A^E6C^/C^E6C^ when applied from the outside (Fig. S4, *B* and *C*). This suggests that this membrane-impermeable reagent can penetrate deep into or through VRAC's pore. This was further substantiated by effects on R8C/R8C and Y9C/Q9C mutants (Fig. S4). Indeed, MTSES has been suggested to permeate LRRC8A/E channels (32), and MTS reagents can also permeate other Cl^−^ channels such as cystic fibrosis transmembrane conductance regulator (33).

Glu-6 is conserved in all LRRC8 isoforms. Similar but less pronounced changes in *P*_I_/*P*_Cl_ as in LRRC8A^E6C^/C^E6C^ were observed when LRRC8A^E6C^ was coexpressed with LRRC8D^E6C^ or LRRC8E^E6C^ (Fig. 3*G*). This result may not be explained exclusively by the mutation of the common LRRC8A subunit because Glu-6 must be mutated in both subunits to change the anion permeability of LRRC8A/C heteromers. *P*_I_/*P*_Cl_ of LRRC8A^E6C^/C and LRRC8A/C^E6C^ heteromers was barely and not significantly different from that of WT heteromers (Table 2). This suggests that the N termini of both LRRC8A and LRRC8C contribute to the permeation pathway of LRRC8A/C channels.

**Table 2:** **ΔE_rev_ and P_I_/P_Cl_ for E6C mutants in LRRC8A/C channels**

Mutants	Δ*E*_rev_	*P*_I_/*P*_Cl_	*n*
	*mV*		
WT/WT	−5.90 ± 0.24	1.29 ± 0.01	5
E6C/E6C	−19.9 ± 1.04	2.29 ± 0.10	7
E6C/WT	−7.31 ± 1.0	1.36 ± 0.05	4
WT/E6C	−7.34 ± 1.7	1.36 ± 0.09	5

### LRRC8 N termini modulate voltage-dependent inactivation

**Figure 4.**
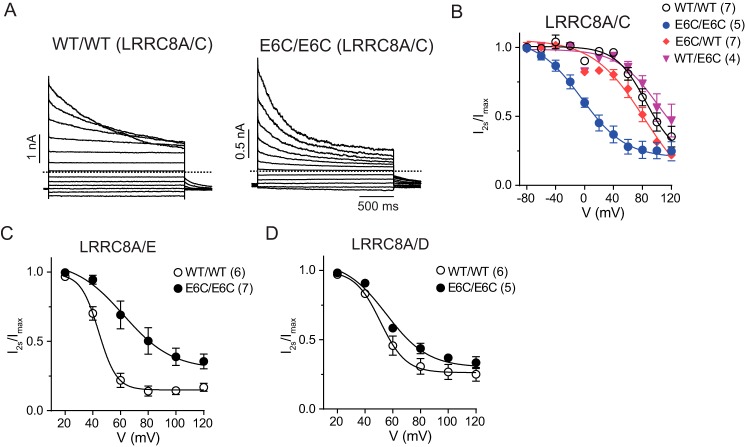
**E6C mutations change inactivation of LRRC8 channels.** *A*, representative current traces of WT/WT (*left*) and E6C/E6C (*right*) LRRC8A/C channels with step protocols as in Fig. 1*B. Dashed lines* indicate zero current. *B*, voltage dependence of inactivation of WT and mutant LRRC8A/C channels. The ratio of currents at the end of the 2-s voltage step (*I*_2s_) and beginning of the pulse (*I*_max_) was fitted by the Boltzmann equation. *C* and *D*, voltage-dependent inactivation of WT/WT and E6C/E6C LRRC8A/E (*C*) and LRRC8A/D (*D*) channels determined as in (*B*). *Error bars*, S.E.; *numbers* in *parentheses*, numbers of replicates.

The permeability-changing E6C double mutation also drastically changed the inactivation of LRRC8A/C heteromers (Fig. 4*A*) by shifting its voltage dependence about 80 mV to more negative potentials (Fig. 4*B*). When inserted in only one of the subunits, these mutations did not affect inactivation (Fig. 4*B*), resembling their lack of effect on ion selectivity (Table 1). E6A/E6A, E6S/E6S, and E6Q/E6Q LRRC8A/C double mutants displayed similar, albeit less pronounced, changes in inactivation (Fig. S3, *A* and *C*). With the caveat that MTSES changed the inactivation of WT LRRC8A/C heteromers, MTSES almost abolished the difference in inactivation between WT/WT and E6C/E6C heteromers, pointing again to a critical role of a negative charge at this position (Fig. S5, *A* and *B*).

The impact of E6C double mutants on inactivation depended on the subunit combination. Whereas a more modest right shift of the voltage dependence of inactivation was observed with LRRC8A/E double mutants (Fig. 4*C*), no effect was seen for LRRC8A/D (Fig. 4*D*).

### N-terminal LRRC8 residues influence current rectification

**Figure 5.**
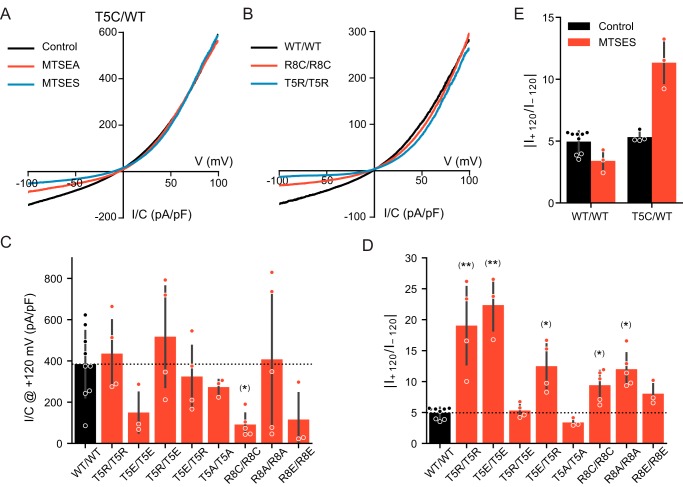
**N-terminal residues affect rectification of LRRC8 channels.** *A*, *I*/*V* curves obtained from ramp protocols from T5C/WT LRRC8A/C channels in the absence or presence of MTSEA or MTSES. *B*, *I*/*V* relationship of WT/WT, T5R/T5R, and R8C/R8C LRRC8A/C channels. *C*, mean *I*_Cl,vol_ densities of the indicated channels at +120 mV. *D*, average rectification defined as the ratio between the maximum current at +120 and −120 mV. *E*, average rectification for WT/WT and T5C/WT LRRC8A/C in the presence or absence of MTSES (the control values for WT the same as in *D*). *Error bars*, S.D.; *, *p* < 0.05; **, *p* < 0.01 (Kruskal–Wallis test, Dunn's post hoc test *versus* WT; false-discovery rate controlled by Benjamini–Hochberg procedure). *pF*, picofarad.

Several charge-modifying mutations in the N terminus or modification of N-terminally inserted cysteines increased the outward rectification of *I*_Cl,vol_. Eliminating the positively charged Arg-8 by mutating it to cysteine or alanine in double mutants increased outward rectification (Fig. 5, *B* and *D*). Likewise, introducing positive charges at Thr-5, either by exposing T5C/WT LRRC8A/C channels to MTSEA or by introducing the T5R mutation in both subunits, increased rectification by selectively decreasing inward currents (Fig. 5, *A*, *B*, and *D*). Of note, introduction of negative charges, by either treating T5C/WT heteromers with MTSES or by introducing T5E mutations into both subunits, also similarly increased rectification (Fig. 5, *E* and *D*). It seems unlikely that increased rectification is due to direct electrostatic interactions with the permeating Cl^−^ ion because it was observed with insertions of either positive or negative charges. We hypothesized that inserting charges of equal signs into all six LRRC8 subunits may rather cause conformational changes of the pore by electrostatic repulsion of their N-terminal segments. Indeed, when we inserted positive charges into one isoform but negative charges into the other isoform as in T5E/T5R and T5R/T5E heteromers, rectification was reduced or restored to WT levels, respectively (Fig. 5*D*).

## Discussion

We identified the short hydrophilic N termini of LRRC8 proteins as major determinants of the biophysical properties of VRAC. Mutations of several of the ∼20 amino acids preceding the first transmembrane domain and chemical modification of N-terminal cysteine mutants not only changed current magnitudes but also inactivation gating and, most importantly, pore properties such as iodide/chloride permeability ratios and rectification. Our data are compatible with a model in which the N termini of LRRC8 subunits line the cytoplasmic portion of the pore, possibly by folding back into the ion translocation pathway.

### Structure and function of VRACs

Structure–function analysis of volume-regulated anion channels is in its infancy as the molecular makeup of VRAC as LRRC8 heteromers has only been discovered recently (4, 15). Based on the homology to pannexins and the presumed similarity to connexins, which unambiguously assemble to hexamers (31), LRRC8 proteins were postulated to form hexamers (16). This hypothesis is compatible with experimental data (6, 17, 34). Although cryo-EM indicated that invertebrate innexin-6, which displays sequence homology to LRRC8 (16), rather displays an octameric pore (26), cryo-EM recently confirmed that LRRC8/VRAC channels are hexamers (27). The ratio of different subunits incorporated into single, physiologically always-heteromeric VRACs appears variable (6, 34). Sequential coimmunoprecipitation experiments showed that native VRACs can contain at least three different LRRC8 isoforms in a single channel (6). Even under the simplified conditions of our experiments (expression of only two LRRC8 isoforms), cells might express many different VRACs that differ not only in the stoichiometry of the two subunits but also in their spatial arrangement. Whole-cell *I*_Cl,vol_ reflects averaged properties of differently composed VRACs.

The LRRC8 isoform composition influences the voltage-dependent inactivation (4, 18), substrate specificity (5, 6, 19), rectification and single channel conductance (17), and sensitivity to oxidation (32) of VRACs. Few studies have used mutagenesis to identify functionally important LRRC8 residues. The LRRC8A T44C mutant slightly increased VRAC's *P*_I_/*P*_Cl_ permeability ratio from 1.29 to 1.59 when expressed in *LRRC8A* knockdown cells (15); LRRC8A^K98E^, when expressed together with the equivalent LRRC8E^E91E^ mutant, slightly decreased *P*_I_/*P*_Cl_ of LRRC8A^K98E^/E^E91E^ channels from 1.25 to 1.12 (18); and LRRC8A^R103A^ increased the cation permeability of LRRC8A^R103A^/C channels (27). Thr-44 is located at the end of TM1, and Lys-98 and Arg-103 are located in EL1. In accord with a recent cryo-EM structure (27), these data suggest that these residues are located close to the extracellular opening of the pore. Mutations in EL1 also drastically affected voltage-dependent inactivation of VRAC (18).

### LRRC8 N termini contribute to the pore of VRACs

**Figure 6.**
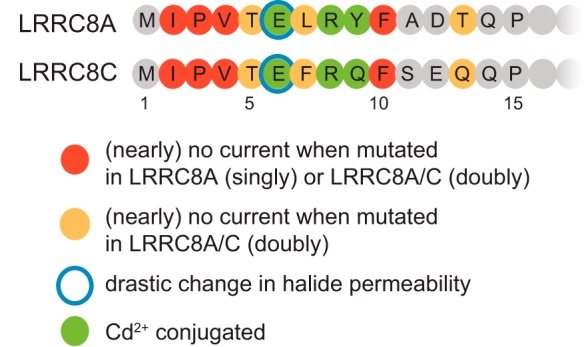
**Summary of functional effects of N-terminal mutations in LRRC8A and LRRC8C.**

The functional importance of LRRC8 N termini first became apparent when fusion of epitopes to the N, but not the C, termini of LRRC8 proteins abolished VRAC currents. Several, but not all, deletions and insertions of N-terminal amino acids also abolished *I*_Cl,vol_ (Figs. 1, *F* and *G*, and 6), and single-cysteine replacements at seven of 14 positions obliterated currents when inserted into both subunits of LRRC8A/C channels (Figs. 2*A* and 6). With one exception (Q14C/Q14C, which is rather close to the first transmembrane span and did not change currents by itself), reaction of functional LRRC8A/C cysteine double mutants with MTSEA reduced or enhanced (R8C) *I*_Cl,vol_ amplitudes (Fig. 2*D*), demonstrating that the mutated residues are both accessible from the aqueous phase and important for channel function. Although it is often inferred from channel inhibition by MTS reagents that the cysteine-substituted residue lines the pore (35–38), alternative interpretations seem possible. However, the inhibition by Cd^2+^ of several cysteine mutants, including the crucial E6C/E6C and R8C/R8C substitutions, requires a close proximity of the respective residues (∼5–9 Å) (39). The Cd^2+^ block of LRRC8A^Y9C^/C heteromers demonstrates that binding does not occur exclusively between N termini of different isoforms. Although we cannot strictly exclude that the introduced cysteines bind Cd^2+^ together with other cytoplasmic cysteines, these results suggest that N termini from different subunits of the hexameric channel come together to form a narrow funnel that likely is part of the channel pore.

Compelling evidence for LRRC8 N termini lining the pore comes from the drastically altered ion permeability of the E6C/E6C mutant. The change in *P*_I_/*P*_Cl_ from ∼1.3 to ∼2.3 by far exceeds those of the T44C (15) and K98E (18) mutants, which change residues close to the extracellular face. The remarkable restoration to WT values of both ion permeability and current amplitudes by negatively charged MTSES but not by positively charged MTSEA shows the importance of a negative charge at position 6. Further strong evidence for a role of LRRC8 N termini in pore formation comes from the changes in current rectification with charge-altering mutations at position 5 or 8 because in VRACs rectification is intrinsic to the pore (40, 41). In conclusion, the analysis of our large set of mutants, summarized in Fig. 6, strongly suggests that LRRC8 N termini contribute to the pore of VRACs.

### Coupling between permeation and inactivation gating

Voltage-dependent inactivation of macroscopic VRAC currents involves the stepwise closure of individual channels that show little gating at voltages not causing inactivation (42). The large difference in inactivation between LRRC8A/C and LRRC8A/E (4) was exploited to identify residues in EL1 as major determinants of inactivation gating (18). Residues in that segment also slightly changed *P*_I_/*P*_Cl_, thereby linking permeation and gating (18). Here, we observed a much stronger link between permeation and gating. LRRC8A/C channels carrying the E6C mutation in both subunits not only displayed strongly altered I^−^/Cl^−^ permeability but also a large shift in the voltage dependence of inactivation to potentials that were even more negative than observed with LRRC8A/E heteromers. MTSES modification suggested that both changes depended on the elimination of the negative charge at position 6. Like inactivation of WT/WT heteromers (4, 18), the effect of E6C mutations also depended on the subunit composition. E6C double mutants led to a marked left shift of voltage-dependent inactivation with LRRC8A/C but a right shift with LRRC8A/E, channels that normally inactivate at very positive and more negative voltages, respectively (4, 18).

Coupling of permeation and gating or inactivation in ion channels is not unprecedented. For instance, constriction of the selectivity filter was implicated in the inactivation of certain potassium channels (43). Permeation and gating are intimately linked in CLC chloride channels (44–46), and the inactivation of native VRAC currents in myoblasts and human embryonic kidney and HL-60 cells has been reported to depend on chloride (47, 48).

### Comparison with pannexin, connexin, and CALHM channels

LRRC8 (VRAC), pannexin, innexins, connexin, and CALHM channels share the same transmembrane topology and have rather large pores that pass not only ions but also organic compounds. Only LRRC8, pannexin, and innexin proteins share sequence similarity (16), but all these channels, each of which belongs to a distinct small gene family, share several other features. Although previously shown only for connexins (31), these channels were believed to be assembled from six identical or homologous proteins (16, 25, 49) as recently confirmed for LRRC8 channels (27). However, *C. elegans* innexins rather form octameric hemichannels in hexadecameric gap junctions (26). Gap junctions are formed by the reciprocal binding of two connexin or innexin “hemichannels” expressed on two closely apposed cells. Connexins can also form isolated hemichannels, whereas pannexin, LRRC8, and CALMH channels do not form gap junctions.

Analysis by both the substituted-cysteine accessibility method (36, 38, 50) and ion permeability–changing substitution (51) and the crystal structure of Cx26 (31) revealed that the second half of TM1 and part of EL1 line the external part of the connexin pore. These structures also influence “loop gating” of connexins (52). The substituted-cysteine accessibility method similarly suggested the end of TM1 and part of EL1 as part of the Panx1 pore (53). These findings are reminiscent of ion permeability–changing mutations in LRRC8A at the end of TM1 (15) and in EL1 (18) and the role of EL1 in VRAC inactivation gating (18).

However, the similarities go much further. As shown here for LRRC8 channels, in connexins the N termini also profoundly affect pore properties as shown by mutations changing ion permeability (54) and rectification (51, 55). Voltage-dependent “*V_j_*” gating of connexins is strongly affected by N-terminal charges, which may sense the electrical field across the membrane (50). Mutations affecting connexin N termini have been identified in various human diseases (56, 57) and stress the importance of that region. The role of N termini in pannexins and CALHM channels is less clear. Cysteine substitutions of several N-terminal Panx1 residues abolished currents (53), whereas exposure of N-terminal functional cysteine mutants to a cysteine modification reagent failed to significantly inhibit currents. Although showing that the N terminus is important, these experiments fall short of demonstrating a role in pore formation. Large parts of the CALHM N terminus can be deleted without loss of currents, which, however, show changed gating (58). Of note, the N termini of connexins, LRRC8 proteins, and most innexins are of almost the same lengths, compatible with similar functions even in the absence of sequence similarity, whereas pannexins, depending on the isoform, have 6–23-residue-longer N termini (16).

The crystal structure of Cx26 revealed that the N termini of the individual subunits fold back and line the cytoplasmic end of the pore (31). Similarly, the N termini of innexins, which show weak homology to LRRC8 proteins (16), dip into the pore (26). Unfortunately, the N termini of LRRC8 proteins are not resolved in the recently published cryo-EM structure (27). Our results are compatible with a similar back-folding of LRRC8 N termini. However, we cannot exclude the possibility that the N termini rather protrude into the cytoplasm where they probably are surrounded by peptide chains from the TM2-TM3 loop and the linker from TM4 to the leucine-rich repeats and provide an intracellular access funnel. Changes in voltage-dependent gating would be somewhat more difficult to explain with the second model because the N termini would not feel the transmembrane voltage.

It should also be noted that, unlike channels with a highly selective, narrow pore (21, 59), VRAC tolerated many different point mutations and deletions in the N terminus without losing its ability to conduct chloride. This relaxed structural requirement, which is also reflected in the lower degree of similarity between LRRC8 N termini compared with transmembrane spans, may be related to the fact that its apparently large pore can also conduct many different organic compounds irrespective of charge (4–6, 34). A rather flexible arrangement of LRRC8 N termini, as suggested by the present data, may explain the inability to resolve them in cryo-EM studies (27).

In conclusion, our work demonstrates that the N termini of LRRC8 subunits, which could not be resolved by cryo-EM (27), participate in forming the cytoplasm-oriented part of VRAC's pore. They may fold back into the permeation pathway as shown for connexins (31) and innexins (26). Permeability and gating of LRRC8 channels are largely determined by their extreme N termini but also by the end of transmembrane domain 1 and the first extracellular loop, resembling findings obtained for connexins.

## Materials and methods

### Molecular biology

Human LRRC8A (NM_019594), LRRC8C (NM_032270), LRRC8D (NM_001134479) and LRRC8E (NM_025061) were used as described before (4, 18). Point mutations were introduced using QuikChange (Agilent) and verified by sequencing the complete ORF.

### Cell culture and transfection

HCT116 *LRRC8*^−/−^ cells (4, 18), with disruption of all five *LRRC8* genes, were maintained in McCoy's 5A medium (PAN Biotech) supplemented with 10% FBS (PAN Biotech) and 1% penicillin/streptomycin at 37 °C and 5% CO_2_. For recording, trypsin-treated cells were seeded onto gelatin-coated coverslips and transfected using Lipofectamine 2000 (Life Technologies). LRRC8C, -D, and -E were fused at the C terminus to GFP or tdTomato and transfected together with LRRC8A at a 1:1 ratio, respectively. Because LRRC8A is needed for the transport of LRRC8B–E to the plasma membrane (4), cell-surface fluorescence of GFP or tdTomato, as observed on the patch-clamp setup, indicated the plasma membrane localization of LRRC8 heteromers. Such cells were used for whole-cell recordings (18–24 h after transfection).

### Immunocytochemistry and antibodies

For immunocytochemistry, cells were fixed in precooled methanol at −20 °C for 15 min 24–36 h after transfection, blocked for 30 min in blocking buffer (PBS containing 0.1% saponin and 3% BSA), and then incubated sequentially for 1 h each with primary and secondary antibodies in blocking buffer. Images were acquired with an LSM 880 confocal microscope with a 63×, 1.4 numerical aperture oil-immersion lens (Zeiss). Rabbit polyclonal antibody against LRRC8A has been described before (4), and GFP antibody was purchased from Aves (GFP-1020). Secondary antibodies were conjugated to Alexa Fluor 488 or 546 (Molecular Probes).

### Whole-cell voltage-clamp recordings

VRAC currents were recorded in the standard whole-cell configuration at room temperature using an EPC-10 patch-clamp amplifier and PatchMaster software (HEKA Elektronik) or MultiClamp 700B patch-clamp amplifier/Digidata 1550B digitizer and pClamp 10 software (Molecular Devices). Patch pipettes were filled with solution containing 40 mm CsCl, 100 mm cesium methanesulfonate, 1 mm MgCl_2_, 1.9 mm CaCl_2_, 5 mm EGTA, 4 mm Na_2_ATP, and 10 mm HEPES (pH 7.2, 290 mosm) and had a resistance of 2–4 megaohms. For Cd^2+^ block experiments, CaCl_2_ and EGTA were omitted from the pipette solution, which then contained 40 mm CsCl, 110 mm cesium methanesulfonate, 1 mm MgCl_2_, 4 mm Na_2_ATP, 10 mm HEPES (pH 7.2, 290 mosm).

The isotonic extracellular solution contained 150 mm NaCl, 6 mm KCl, 1 mm MgCl_2_, 1.5 mm CaCl_2_, 10 mm glucose, and 10 mm HEPES (pH 7.4, 320 mosm). To elicit I_Cl,vol_, cells were exposed to a 25% hypotonic solution containing105 mm NaCl, 6 mm CsCl, 1 mm MgCl_2_, 1.5 mm CaCl_2_, 10 mm glucose, 10 mm HEPES (pH 7.4, 240 mosm). Cesium was used instead of potassium in the hypotonic external solution and the pipette solution to block potential potassium current, which could be activated during cell swelling. For measuring ion selectivity, NaCl in the hypotonic solution was substituted with equimolar amounts of NaI, NaSCN, NaBr, or NaF. To record I_Cl,vol_, the standard protocol consisted of a 0.6-s step to −80 mV followed by a 2.6-s ramp from −100 to +100 mV from a holding potential of −30 mV applied at 15-s intervals (as shown in Fig. 2*B*). Currents at −80 mV were used to analyze current density. To examine the inactivation and rectification of VRAC, voltage protocols consisted of a 2-s step protocol from −120 to +120 mV in 20-mV increment from a holding potential of −80 mV applied every 5 s (illustrated in Fig. 1*B*). Recordings were low pass–filtered at 2 kHz and sampled at 20 kHz.

### Reagents and chemical modification

MTSEA and MTSES were purchased from Biotium and stored at −20 °C as powder. Stock solutions of MTSEA and MTSES were freshly made every day and kept at −20 °C. Solution containing 200 μm MTSEA or 1 mm MTSES was freshly prepared. All other reagents were bought from Sigma-Aldrich.

### Data analysis

Relative anion permeabilities (*P_X_*^−^/*P*_Cl_^−^) were calculated from the shifts of reversal potential using a modified Goldman–Hodgkin–Katz equation,

(Eq. 1) $$\frac{P_{X}}{P_{\text{Cl}}}=\frac{{\left[\text{Cl}\right]}_{\text{hypo}}e^{\left(-\Delta E_{\text{rev}}F/RT\right)}-{\left[\text{Cl}\right]}_{\text{subst}}}{{\left[X\right]}_{\text{subst}}}$$

where Δ*E*_rev_ is the shift in reversal potential, [Cl]_hypo_ and [Cl]_subst_ are the extracellular Cl^−^ concentrations in the normal and anion-substituted hypotonic saline (I^−^, SCN^−^, F^−^, and Br^−^), and [*X*]_subst_ is the concentration of the substituting anion. *R* is the gas constant, *T* is the absolute temperature, and *F* is the Faraday constant. Effects of acute MTSEA or MTSES application were assessed as the ratio of current amplitude at the end of application over steady-state current in hypotonic medium immediately before application.

Liquid junction potentials were measured for all solutions and corrected for in ion selectivity experiments. *V*_½_ for inactivation was calculated as described (18). Because inactivation was generally too slow for currents to reach steady state, we used the ratio of currents at the end of the 2-s voltage step (*I*_2s_) by the maximal current amplitude at the beginning of the voltage step (*I*_max_) as the measure for inactivation. The time of half-inactivation *t*_½_ was defined as the time point where the inactivation reached half of the inactivation after 2 s. Boltzmann curve fitting and calculation of *V*_½_ were performed using GraphPad Prism with the following fitting constraints: bottom value less than 0.2 and top value greater than 0.9. Statistical analysis was conducted with GraphPad Prism and SciPy library for Python programming language (Python Software Foundation). For pairwise comparisons of current densities and anion permeability ratios the Mann–Whitney test and Welch *t* test, respectively, were used to evaluate statistical significance. The obtained *p* values were corrected for multiple comparisons using the Benjamini–Hochberg procedure to control the false-discovery rate when appropriate. Kruskal–Wallis test was used for comparing multiple groups with Dunn's post hoc test for pairwise comparisons.

## Author contributions

P. Z., M. M. P., and T. J. J. validation; P. Z., M. M. P., and T. J. J. investigation; P. Z. methodology; P. Z. and T. J. J. writing-original draft; M. M. P. and T. J. J. writing-review and editing; T. J. J. conceptualization; T. J. J. supervision; T. J. J. funding acquisition.

## Supplementary Material

Supporting Information

## Footnotes

4 The abbreviations used are:

VRAC: volume-regulated anion channel
LRR: leucine-rich repeat
LRRC8: leucine-rich repeat–containing 8
CALHM: calcium homeostasis modulator
EL1: first extracellular loop
MTS: methanethiosulfonate
MTSEA: 2-aminoethyl methanethiosulfonate
TM: transmembrane domain
MTSES: 2-sulfonatoethyl methanethiosulfonate
*P*: permeability
Δ*E*_rev_: shift of reversal potential.
