# Supplementary material for: LRRC8 N termini influence pore properties and gating of volume-regulated anion channels (VRACs)
Source: J Biol Chem. 2018 Jun 20;293(35):13440–51. doi: 10.1074/jbc.RA118.002853 (PMC6120214; doi:10.1074/jbc.RA118.002853)
Supplement: Supporting Information [file supp_293_35_13440__index.html]

LRRC8 amino-termini influence pore properties and gating of volume-regulated VRAC anion channels — LRRC8 N-termini in VRAC function — LRRC8 N termini influence pore properties and gating of volume-regulated anion channels (VRACs) — LRRC8 N termini in VRAC function — Supporting Information 

# LRRC8 N termini influence pore properties and gating of volume-regulated anion channels (VRACs)

## Supporting Information

- Supporting Information for Zhou, Polovitskaya, Jentsch - Supplementary Figures
