# Supplementary material for: LRRC8 N termini influence pore properties and gating of volume-regulated anion channels (VRACs)
Source: J Biol Chem. 2018 Jun 20;293(35):13440–51. doi: 10.1074/jbc.RA118.002853 (PMC6120214; doi:10.1074/jbc.RA118.002853)
Supplement: Supporting Information [file supp_RA118.002853_136600_2_supp_156266_pskfs6.pdf]

Supporting Information for Zhou, Polovitskaya & Jentsch

Supplementary Figures

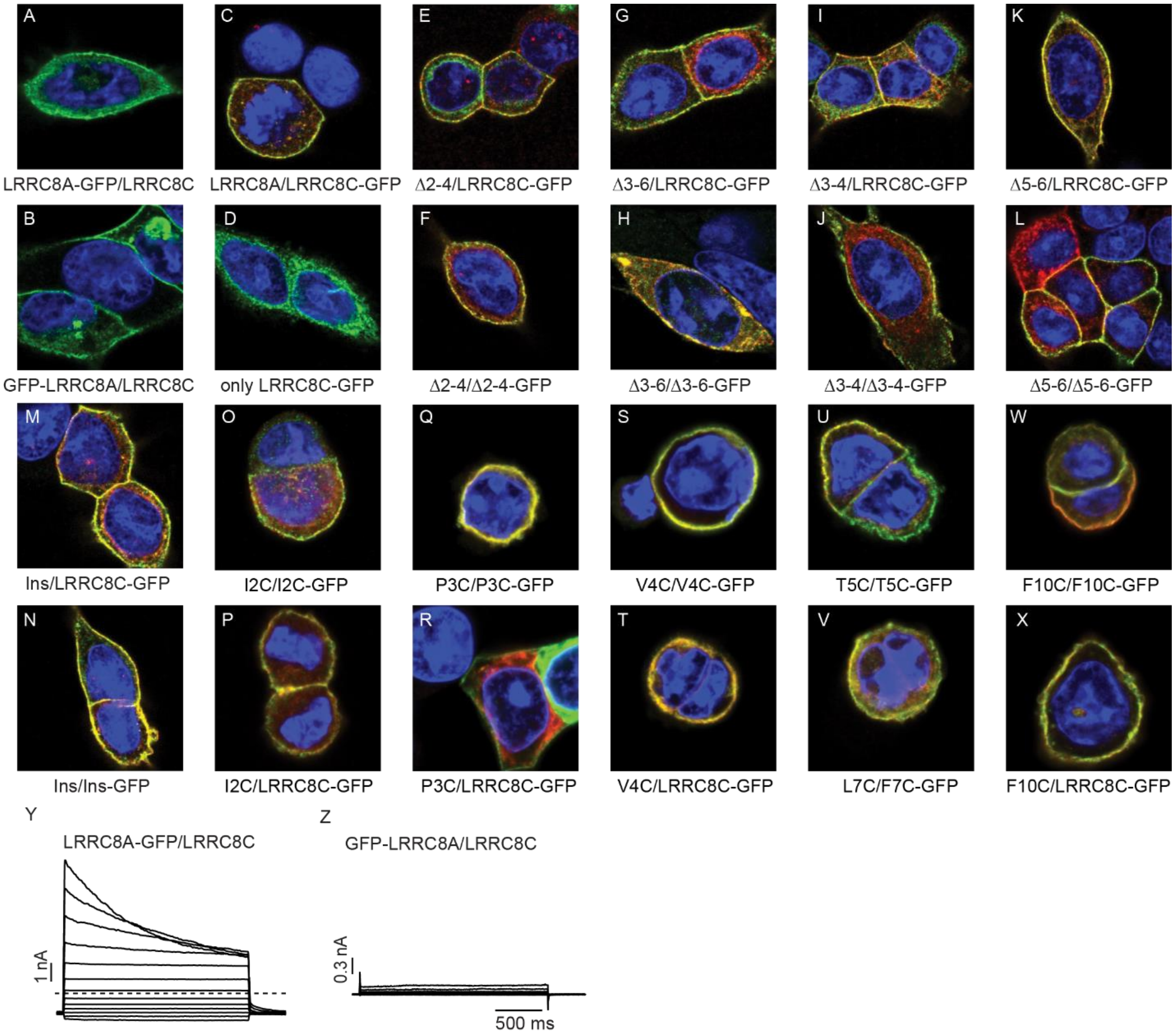

Figure S1. Plasma membrane localization of amino-terminal mutants of LRRC8A/C heteromers and effect of GFP fusion on $I_{Cl,vol}$ currents. *A*-*N*, Immunofluorescent images of *LRRC8*$^{-/-}$ HCT116 cells transiently transfected with LRRC8A- and -C encoding plasmids, with subunits fused to GFP and carrying mutations as indicated. *A*,*B*, LRRC8A (GFP signal) localized to the plasma membrane irrespective of whether GFP was attached to the C-terminus (*A*) or N-terminus (*B*). *C*,*D*, LRRC8C-GFP reached plasma membrane when co-transfected with LRRC8A (*C*), whereas without co-transfection of LRRC8A, GFP-tagged LRRC8C localizes to the ER (*D*) as described previously (4), as revealed by anti-GFP (green) and anti-LRRC8A (red) antibody labeling. The image in panel C is the same as shown in Fig 1A. *E*-*X*, Together with LRRC8A, LRRC8C-GFP reaches the plasma membrane, irrespective of deletions, insertions or cysteine mutants in LRRC8A or LRRC8C. Red: anti-LRRC8A; green: anti-GFP. Yellow color results from superimposed green and red signals, and indicates the co-localization of both subunits in LRRC8A/C heteromers. *Y*,*Z*, $I_{Cl,vol}$ could be elicited from LRRC8A/C heteromers if GFP was fused to the C-terminus of LRRC8A (*Y*), but not when it was attached to its N-terminus (*Z*). Currents measured as in Fig. 1*B*.

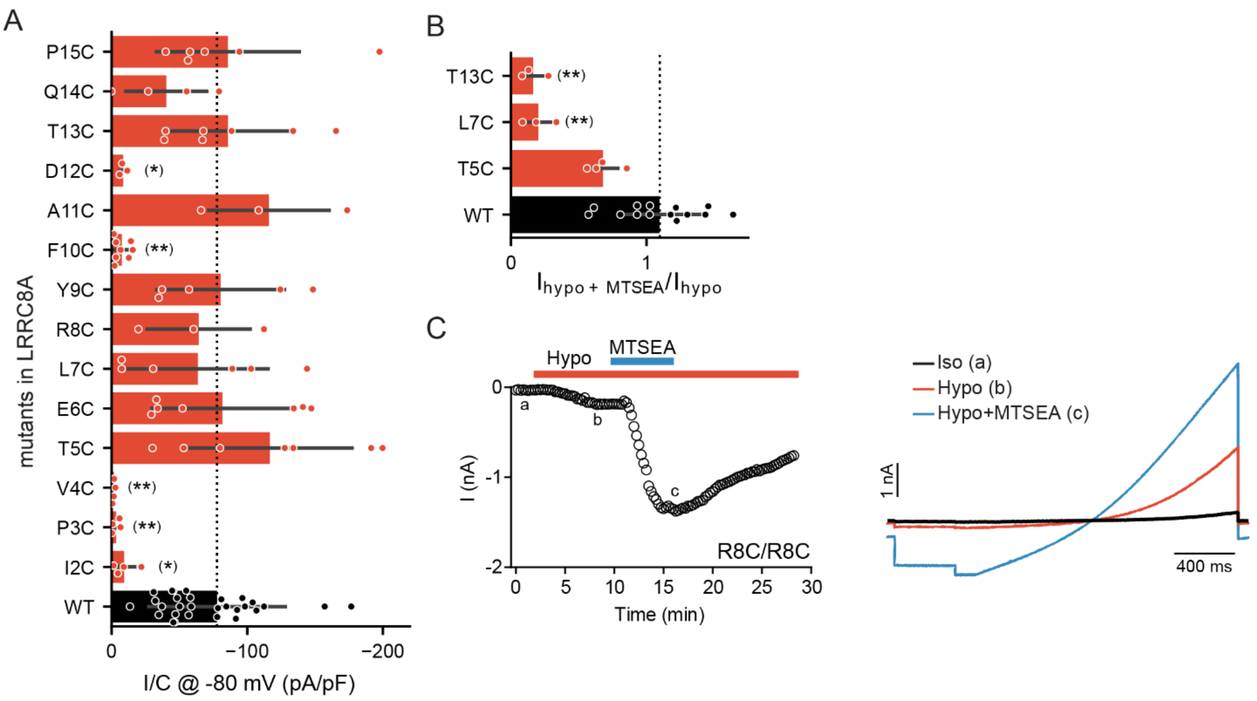

Figure S2. Cysteine scanning analysis of LRRC8A in heteromers with WT LRRC8C, and potentiation of R8C/R8C mutant LRRC8A/C channels by MTSEA. *A*, Mean maximum $I_{Cl,vol}$ current densities of LRRC8A/C channels carrying cysteine substitutions only in LRRC8A (the values for WT are the same as in Fig. 1*F*). *B,* Mean effect of MTSEA on maximal $I_{Cl,vol}$ currents at −80 mV of LRRC8A/C heteromers carrying cysteine mutations in only LRRC8A (the values for WT are the same as in Fig. 2*D*). *C*, Effect of MTSEA on LRRC8A/C carrying the R8C mutation in both subunits. Left: Representative time course of the effect of 200 µM MTSEA on $I_{Cl,vol}$ at −80 mV, obtained from ramps as shown at right, in isotonic (a), hypotonic (b), hypotonic plus MTSEA (c) solutions. Error bars, standard deviation; * $p < 0.05$, ** $p < 0.01$ in *A*, *C* versus WT; in *A* and *C*, Kruskal-Wallis test, Dunn's post hoc test; false-discovery rate controlled by Benjamimi-Hochberg procedure.

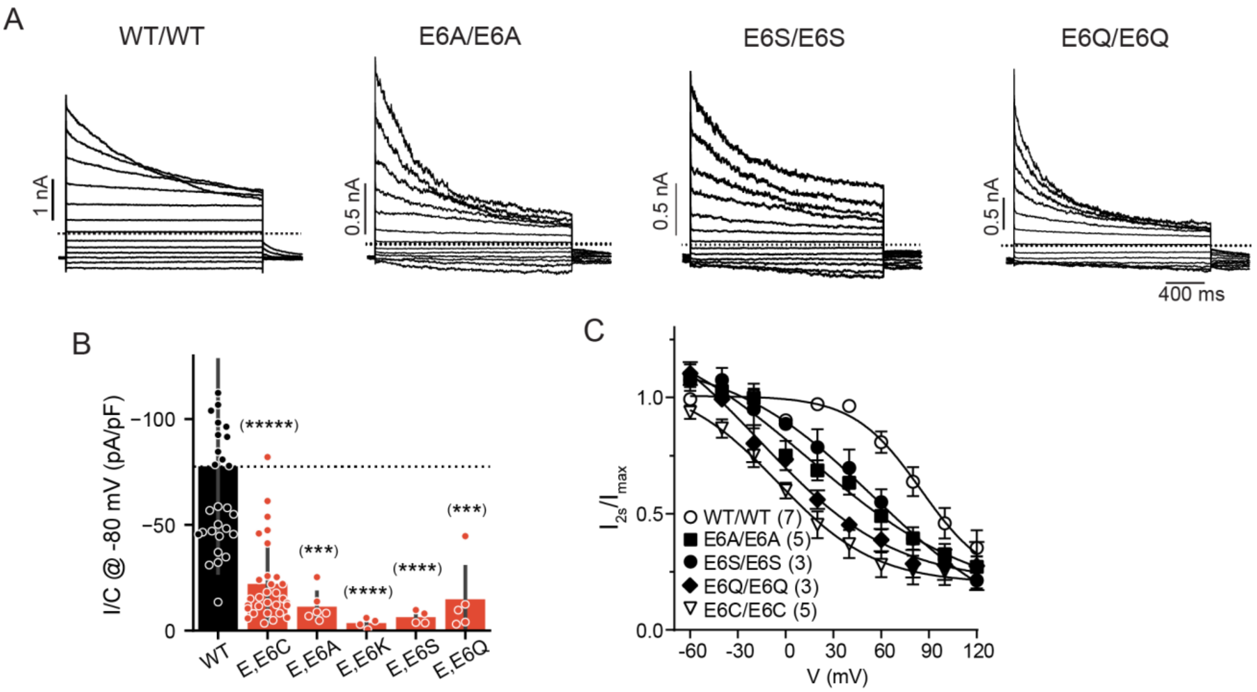

Figure S3. Mutations at E6 change conductance and inactivation gating of LRRC8A/C channels. *A*, Typical current traces for WT/WT, E6A/E6A, E6S/E6S and E6Q/E6Q mutants of LRRC8A/C channels. Dotted lines, zero current levels. *B*, Mean $I_{Cl,vol}$ current densities at −80 mV for indicated LRRC8A/C heteromers (the values for WT are the same as in Fig. 1*F*). *C*, Voltage-dependence of inactivation determined as in Fig. 4 for WT and corresponding mutants as indicated. Error bars, standard deviation in *B*, s.e.m in *C*; *** p < 0.001, **** p < 0.0001 versus WT (Kruskal-Wallis test, Dunn's post hoc test, false-discovery rate controlled by Benjamimi-Hochberg procedure), cell numbers in parentheses.

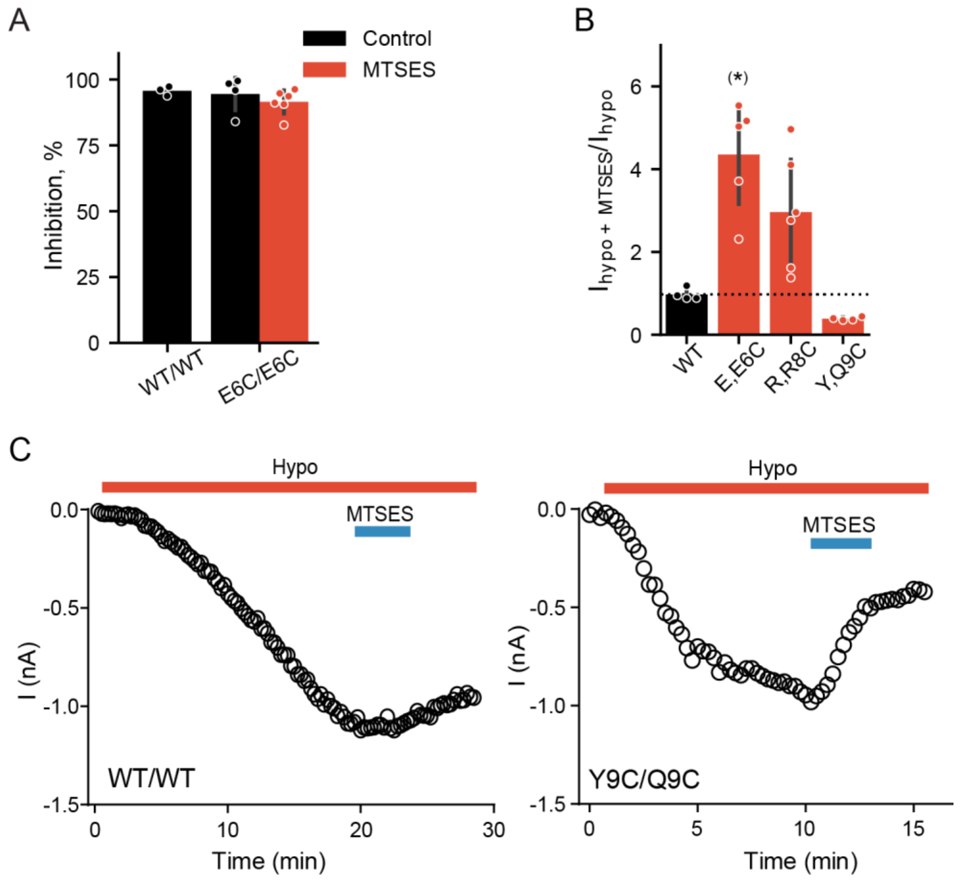

Figure S4. Effect of MTSES on LRRC8A/C mutants. *A*, Inhibition of $I_{Cl,vol}$ by 20 µM DCPIB in WT/WT channels, and in E6C/E6C in presence and absence of 1 mM MTSES in pipette solution. *B*, Effect of extracellular MTSES (1 mM) on WT/WT, E6C/E6C, R8C/R8C and Y9C/Q9C LRRC8A/C channels. *C*, Typical time of $I_{Cl,vol}$ of WT/WT (left) and Y9C/Q9C (right) LRRC8A/C channels as stimulated by 25% hypotonic solution (red bar) and in response to 1 mM MTSES (blue bar) applied in extracellular solution in continued presence of hypotonicity. Error bars, standard deviation; * $p < 0.05$ versus WT (Kruskal-Wallis test, Dunn's post hoc test, false-discovery rate controlled by Benjamimi-Hochberg procedure).

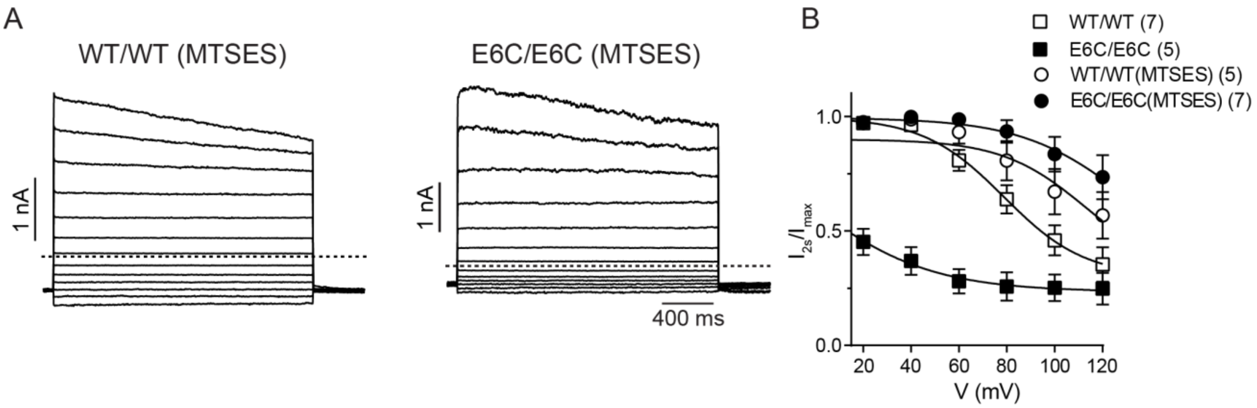

Figure S5. Inactivation gating of WT and E6C mutant LRRC8A/C channels after exposure to MTSES. *A*, Representative I-V curves for WT/WT and E6C/E6C in LRRC8A/C heteromers with 1 mM MTSES in pipette solution. *B*, Inactivation curves (obtained as in Fig 4) for indicated combinations with MTSES in intracellular solutions. No significant difference of $V_{1/2}$ between WT/WT and E6C/E6C with MTSES. Error bars, s.e.m in *B*, standard deviation in *C*. Cell numbers indicated in parenthesis.
